# Carbon sequestration and credit potential of gamhar (*Gmelina arborea* Roxb.) based agroforestry system for zero carbon emission of India

**DOI:** 10.1038/s41598-024-53162-5

**Published:** 2024-02-28

**Authors:** Abhay Kumar, M. S. Malik, Swati Shabnam, Rakesh Kumar, S. Karmakar, Soumitra Sankar Das, Kerobim Lakra, Indra Singh, Rikesh Kumar, Asha Kumari Sinha, Sheela Barla, Nargis Kumari, P. R. Oraon, Muneshwar Prasad, Wajid Hasan, Dinesh Mahto, Jeetendra Kumar

**Affiliations:** 1 KVK, Jehanabad, Bihar Agricultural University, Sabour, Bhagalpur, Bihar 813210 India; 2 Faculty of Forestry, Birsa Agricultural University, Ranchi, Jharkhand 834006 India; 3 Department of Soil Science and Agricultural Chemistry, Ranchi Agriculture College, Birsa Agricultural University, Ranchi, Jharkhand 834006 India; 4 Department of Agronomy, Ranchi Agriculture College, Birsa Agricultural University, Ranchi, Jharkhand 834006 India; 5 Faculty of Management and Commerce, The ICFAI University, Agartala, Tripura 799210 India; 6 Department of Agricultural Economics, Ranchi Agriculture College, Birsa Agricultural University, Ranchi, Jharkhand 834006 India; 7 Department of Silviculture, VCSG Uttarakhand University of Horticulture and Forestry, Bharsar, Uttarakhand 246123 India; 8 Institute of Forest Productivity, Ranchi, Jharkhand 835303 India

**Keywords:** Climate sciences, Climate change, Environmental sciences, Environmental impact

## Abstract

The agroforestry system is the best option to achieve the net zero carbon emissions target for India. Keeping this view, carbon sequestration and credit potential of gamhar based agroforestry system has been assessed. The experiment was carried out in randomized block design in seven different treatments with five replications. Gamhar tree biomass accumulation was higher in gamhar based agroforestry system compared to sole gamhar. Among different tree components, stem contributed a maximum to total gamhar tree biomass followed by roots, leaves and branches. The average contributions of stems, roots, leaves and branches in total tree biomass in two annual cycles (2016–17 and 2017–18) varied between 50 and 60, 19.8 and 20, 19.2 and 20, and 10.7 and 12.7 percent, respectively. In case of crops, above ground, below ground and total biomass was significantly higher in sole intercrops than gamhar based agroforestry system. Total (Tree + interrops + Soil) carbon stock, carbon sequestration, carbon credit and carbon price were significantly affected by treatments, and was maximum in Sole Greengram-Mustard. Net carbon emission was also recorded lowest in Sole Greengram-Mustard for which the values were 811.55% and 725.24% and 760.69% lower than Sole Gamhar in 2016–17, 2017–18 and in pooled data, respectively.

## Introduction

In recent decades, carbon management is an important point on the agenda to identify the best viable mitigation strategies for its reduction^1^. The total greenhouse gases (GHGs) emissions increased almost linearly from 746.5 Mt CO_2_e in 1970 to 3375 Mt CO_2_e in 2018^2^. Presently, India is the 3rd major country in worldwide energy use and anthropogenic emissions of carbon, after China and United States of America, of which energy sector contributes 75 percent (2129 Mt CO_2_e) of overall carbon emissions^3^. At COP26 held in 2021, Glasgow, United Kingdom, Prime Minister of India announced a net zero carbon emissions target by 2070 and proposed a ‘One-Word Movement’ to the global community i.e., L I F E…Lifestyle for Environment as lifestyle has a big role in climate change. For this he has given five strategies called *Panchamrit* (Achieving net zero carbon by 2070; Reducing carbon intensity upto 45 percent by 2030; 50 percent energy requirement to be met by renewable sources by 2030; Generate 500 GW energy from non-fossil fuel by 2030 and Reduce 1billion tons carbon emissions by 2030). Two out of these are short term targets that would cover the way for targeting a net zero carbon emissions goal by 2070. The instant targets are reducing 1 bt carbon emissions by 2030 and reducing carbon intensity below 45 percent by 2030 at 2005 level. Agroforestry has emerged as a strategy for climate change mitigation by reducing greenhouse gases emission through sequestering carbon^4^.

Due to climate change, losses equivalent to at least 5% of global GDP each year and a possibility of 10–40 percent loss in crop production in India due to floods and droughts are anticipated. Population pressure, agricultural expansion/intensification, deforestation and development of infrastructure have been the major threats to biodiversity and climate^5^. In the wake of climate change and declining factor productivity, all governments are supporting agroforestry system due to its role in soil health improvement, nutrient cycling, carbon sequestration and better economic returns as compared to existing cropping systems posing more pressure on natural resources. Agroforestry systems have been identified as a climate change adaptation strategy by 29 countries including India, while 23 countries have identified it as a mitigation strategy in their Intended Nationally Determined Contributions (INDCs) under the UNFCCC^6^. Agroforestry has significant potential to contribute to 9 out of the 17 Sustainable Development Goals^7^. United State Development Authority developed a planned agenda for agroforestry for the period of 2011–16 in 2011, to strengthen research and extension in agroforestry. The 2022 edition of The State of the World’s Forests explores the potential of three forest pathways for achieving green recovery and tackling environmental crises, including climate change and biodiversity loss. These pathways are interrelated, i.e. stopping or reducing deforestation and maintaining forests; restoring degraded lands and expanding agroforestry; and sustainably using forests and building green value chains^8^. India also emphasized the role of agroforestry in sustainable development, starting important policies like Green India Mission, 2010; National Mission for Sustainable Agriculture (NMSA), 2014; National Agroforestry Policy, 2014; Sub-Mission on Agroforestry (SMAF), 2016; National Forest Policy 2018 (Draft); Restructured National Bamboo Mission 2018 (Draft); Indian Forest (Amendment) Act, 2019 (Draft); Forest (Conservation) Rules, 2022 etc. Trees Outside Forests in India (TOFI) is a joint initiative of India and the United States to increase green cover outside forest lands in India. It was jointly launched on September 8, 2022, by the United States Agency for International Development (USAID) and the Ministry of Environment, Forests and Climate Change (MoEFCC) of the Government of India. It aims to expand tree coverage by 2.8 mha through agroforestry, enhance 420 mt carbon dioxide equivalent (CO_2_-eq) sequestration, benefit of 13.1 m people through improving livelihood and environmental services, support local peoples, and strengthen the climate resilience agriculture.

In this context, agroforestry systems sequester a huge amount of carbon in above ground as well as below ground biomass, and soil carbon, regulating the carbon cycle and it is reported that more carbon is stored in different components of agroforestry system compared to conventional plantations, resulting in lower atmospheric greenhouse gas (GHG) concentrations^9,10^. Further, it also reported around 30–45 percent higher carbon sequestered in tree biomass by agroforestry in comparison with natural forest in central Himalaya. Agroforestry system holds mitigation potential of 1.1–2.2 Pg C in terrestrial ecosystems over the next 50 years^11^. Additionally, 630 million ha of unproductive croplands and grasslands could be converted to agroforestry systems with an estimated carbon sequestration potential of 1.43 and 2.15 Tg (1 Tg = 1012 g) CO_2_ annually by 2010 and 2040 respectively^12^. The C sequestration potential of tropical agroforestry systems in recent studies is estimated between 12 and 228 Mg ha^-1^, with a median value of 95 Mg ha^-1^. The agroforestry practices in Ethiopia, Africa, have resulted in sequestering 8.34–43.64 Mg ha^-1^of carbon in trees and 71.69–112.74 Mg ha^-1^ of carbon in soil^13^. Further, it is also reported that at 5 years *Gmelina*, total stand biomass in agrisilviculture system was 14.1 Mg ha^-1^. Plantations had 35% higher biomass than agrisilviculture system^14^. The leaves, stem, branches and roots contributed 4.1, 65.2, 10.0 and 20.70%, respectively to total standing biomass (17.9 Mg ha^-1^). He also reported that the rate of SCS was 0.42 Mg C ha^-1^ yr^-1^ in *Gmelina arborea* (576 trees ha^-1^) based agroforestry system. Kumar et al.^15^ reported that the biomass estimates revealed that all tree components viz., leaf, stem, branch and root varied significantly (*p* 0.05) in different tree spacing. Total biomass in five-year-old stands ranged from 6.96 to 13.75 Mg ha^-1^. The contribution of different components for total biomass was in the order of stem > roots > branches > leaves. Stem wood accounted to maximum ranging between 58.4 and 59.7% to total biomass followed by roots (17.5–17.8%), branches (15.9–17.0%) and leaves (6.4–7.1%).

Thus, agroforestry research and development are a broad area of study for enhancing farm economics, employment generation, decreasing poverty, achieving zero hunger, food security, and climate change mitigation, reducing GHGs emission, increasing greenery, and new evergreen revolution etc. Keeping the current global scenario in view, this experiment was planned to assess the role of gamhar based agroforestry system in recent carbon pricing and trading for environmental as well as monetary benefits for the farmers. There is a great need to identify the suitable agricultural crops, which can grow well along with tree species with limited solar energy available underneath the trees. In the present investigation on gamhar woody perennial tree with the intention of growing agricultural crops viz., Arhar (*Cajanus cajan*), Cowpea (*Vigna unguiculata*), Greengram (*Vigna radiata*) and Mustard (*Brassica juncea*) were intercropped. These crops were selected based on their national demand, adaptation, growing habit, production and requirement. Therefore, in this study, also fulfilled the knowledge gap between researchers to farmers for carbon credit is then sold as voluntary emission offsets on the carbon market.

## Results

### Estimation of standing tree biomass carbon stock, sequestration, credit and price

The biomass accumulation was higher in gamhar based agroforestry system compared to sole gamhar (Table 1). Among different tree components, the stem contributed maximum to total tree biomass followed by roots, leaves and minimum by branches. Average contribution of stem, roots, leaves and branches in total tree biomass was 50.00, 19.79, 19.18 and 10.73 percent in 2016–17, 59.94, 19.98, 12.74 and 7.27 percent in 2017–18, and 58.05, 20.00, 14.02 and 7.93 percent in pooled data, respectively.

**Table 1:** Stem, branch, leaf and root biomass of gamhar tree under gamhar based agroforestry system.

Treatments	Stem biomass(t ha^−1^)			Branch biomass(t ha^−1^)			Leaf biomass(t ha^−1^)			Root biomass(t ha^−1^)		
	2016–17	2017–18	Pooled	2016–17	2017–18	Pooled	2016–17	2017–18	Pooled	2016–17	2017–18	Pooled
Gamhar + Arhar	0.085 ± 0.001	0.423 ± 0.020	0.254 ± 0.187	0.019 ± 0.002	0.051 ± 0.001	0.035 ± 0.018	0.037 ± 0.001	0.086 ± 0.006	0.062 ± 0.027	0.035 ± 0.001	0.140 ± 0.004	0.088 ± 0.058
Gamhar + Cowpea-Mustard	0.083 ± 0.002	0.440 ± 0.011	0.262 ± 0.196	0.018 ± 0.000	0.052 ± 0.000	0.035 ± 0.019	0.033 ± 0.003	0.093 ± 0.001	0.063 ± 0.033	0.033 ± 0.001	0.146 ± 0.003	0.090 ± 0.062
Gamhar + Greengram-Mustard	0.085 ± 0.000	0.441 ± 0.008	0.263 ± 0.197	0.017 ± 0.000	0.053 ± 0.001	0.035 ± 0.019	0.031 ± 0.002	0.097 ± 0.001	0.064 ± 0.037	0.033 ± 0.000	0.148 ± 0.002	0.090 ± 0.063
Sole Gamhar	0.078 ± 0.001	0.385 ± 0.009	0.231 ± 0.168	0.017 ± 0.001	0.049 ± 0.001	0.033 ± 0.018	0.026 ± 0.001	0.083 ± 0.002	0.055 ± 0.031	0.030 ± 0.000	0.129 ± 0.002	0.080 ± 0.054
*Sole Arhar	–	–	–	–	–	–	–	–	–	–	–	–
*Sole Cowpea-Mustard	–	–	–	–	–	–	–	–	–	–	–	–
*Sole Greengram-Mustard	–	–	–	–	–	–	–	–	–	–	–	–
SEm ±	0.012	0.039	0.020	0.000	0.005	0.002	0.003	0.012	0.006	0.003	0.008	0.004
CD (*p* = 0.05)	NS	NS	NS	NS	NS	NS	NS	NS	NS	NS	NS	NS
CV (%)	25.658	16.092	19.939	3.672	16.757	17.624	19.419	23.794	25.856	15.047	10.183	12.345

*Sole Arhar, Cowpea-Mustard and Greengram-Mustard not included in statistical analysis.

The results reveal that above ground, below ground as well as total biomass production were not affected by treatments (Table 2). During the first year (2016–17) of experimentation, above ground, below ground and total biomass production was maximum in Gamhar + Arhar which was 16.53, 16.67 and 16.45 percent higher than Sole Gamhar, respectively in 2016–17. During 2017–18 and in pooled data, maximum above ground, below ground and total biomass was recorded in Gamhar + Greengram-Mustard, being 14.31, 14.73 and 14.22 higher than Sole Gamhar in 2017–18 and 13.48, 12.50 and 13.53 percent higher than Sole Gamhar in pooled data, respectively.

**Table 2:** Above ground, below ground and total biomass of gamhar tree under gamhar based agroforestry system.

Treatments	Above ground biomass (t ha^−1^)			Below ground biomass (t ha^−1^)			Total biomass (t ha^−1^)			Carbon stock (t ha^−1^)			Carbon sequestration (t ha^−1^)		
	2016–17	2017–18	Pooled	2016–17	2017–18	Pooled	2016–17	2017–18	Pooled	2016–17	2017–18	Pooled	2016–17	2017–18	Pooled
Gamhar + Arhar	0.141 ± 0.002	0.56 ± 0.017	0.35 ± 0.231	0.035 ± 0.001	0.14 ± 0.004	0.088 ± 0.058	0.177 ± 0.003	0.701 ± 0.022	0.439 ± 0.289	0.088 ± 0.001	0.35 ± 0.011	0.219 ± 0.144	0.324 ± 0.005	1.285 ± 0.040	0.805 ± 0.529
Gamhar + Cowpea-Mustard	0.134 ± 0.004	0.585 ± 0.010	0.359 ± 0.247	0.033 ± 0.001	0.146 ± 0.003	0.09 ± 0.062	0.160. ± 0.006	0.731 ± 0.013	0.449 ± 0.309	0.084 ± 0.003	0.366 ± 0.006	0.225 ± 0.155	0.307 ± 0.010	1.341 ± 0.024	0.824 ± 0.567
Gamhar + Greengram-Mustard	0.133 ± 0.002	0.591 ± 0.007	0.362 ± 0.251	0.033 ± 0.000	0.148 ± 0.002	0.09 ± 0.063	0.166 ± 0.002	0.739 ± 0.008	0.453 ± 0.314	0.083 ± 0.001	0.369 ± 0.004	0.226 ± 0.157	0.305 ± 0.004	1.356 ± 0.015	0.83 ± 0.576
Sole Gamhar	0.121 ± 0.002	0.517 ± 0.008	0.319 ± 0.217	0.03 ± 0.000	0.129 ± 0.002	0.08 ± 0.054	0.152 ± 0.002	0.647 ± 0.010	0.399 ± 0.271	0.076 ± 0.001	0.324 ± 0.005	0.199 ± 0.136	0.278 ± 0.004	1.187 ± 0.018	0.733 ± 0.498
*Sole Arhar	–	–	–	–	–	–	–	–	–	–	–	–	–	–	–
*Sole Cowpea-Mustard	–	–	–	–	–	–	–	–	–	–	–	–	–	–	–
*Sole Greengram-Mustard	–	–	–	–	–	–	–	–	–	–	–	–	–	–	–
SEm ±	0.007	0.024	0.014	0.003	0.008	0.004	0.014	0.041	0.022	0.007	0.021	0.011	0.026	0.076	0.04
CD (*p* = 0.05)	NS	NS	NS	NS	NS	NS	NS	NS	NS	NS	NS	NS	NS	NS	NS
CV (%)	12.2	11.58	11.91	15.047	10.183	12.345	15.057	10.184	12.34	15.034	10.178	12.341	15.063	10.181	12.343

*Sole Arhar, Cowpea-Mustard and Greengram-Mustard not included in statistical analysis.

Similar trend was recorded in case of carbon stock and carbon sequestration where, maximum was recorded in Gamhar + Arhar which was 15.79 and 16.55 percent higher than Sole Gamhar, respectively in 2016–17. However, during 2017–18 and in pooled data, it was maximum in Gamhar + Greengram-Mustard, which was 13.89 and 14.24 percent higher than Sole Gamhar in 2017–18 and 13.57 and 13.23 percent higher than Sole Gamhar in pooled data, respectively.

In the year 2016–17, carbon credit and carbon price were maximum in Gamhar + Arhar which was 16.54 and 16.50 percent higher than Sole Gamhar, respectively. However, during 2017–18 and in pooled data, carbon credit and carbon price was maximum in Gamhar + Greengram-Mustard, which was 14.49 and 14.20 percent higher than Sole Gamhar in 2017–18 and 13.23 and 13.27 percent higher than Sole Gamhar in pooled data, respectively which is presented in Table 3.

**Table 3:** Carbon credit and Carbon price of gamhar tree under gamhar based agroforestry system.

Treatments	Carbon credit (No unit)			Carbon price ($)		
	2016–17	2017–18	Pooled	2016–17	2017–18	Pooled
Gamhar + Arhar	0.324 ± 0.01	1.285 ± 0.04	0.805 ± 0.530	6.48 ± 0.01	25.70 ± 0.04	16.10 ± 0.530
Gamhar + Cowpea-Mustard	0.307 ± 0.01	1.341 ± 0.02	0.824 ± 0.570	6.14 ± 0.01	26.82 ± 0.02	16.48 ± 0.570
Gamhar + Greengram-Mustard	0.309 ± 0.00	1.359 ± 0.02	0.830 ± 0.580	6.18 ± 0.00	27.18 ± 0.02	16.60 ± 0.580
Sole Gamhar	0.278 ± 0.00	1.187 ± 0.02	0.733 ± 0.501	5.56 ± 0.00	23.74 ± 0.02	14.66 ± 0.501
*Sole Arhar	–	–	–	–	–	–
*Sole Cowpea-Mustard	–	–	–	–	–	–
*Sole Greengram-Mustard	–	–	–	–	–	–
SEm ±	0.026	0.076	0.040	0.520	1.520	0.800
CD (*p* = 0.05)	NS	NS	NS	NS	NS	NS
CV (%)	15.063	10.180	12.343	15.051	10.171	12.332

*Sole Arhar, Cowpea-Mustard and Greengram-Mustard not included in statistical analysis.

### Estimation of crops biomass, carbon stock, sequestration, credit and price

Above ground biomass, total biomass as well as carbon stock was maximum in Sole Greengram-Mustard which was 78.67, 95.00 and 87.25 percent higher than Gamhar + Arhar in 2016–17, 77.46, 98.63 and 88.41 percent in 2017–18 and 77.81, 98.89 and 88.69 percent in pooled data, respectively (Table 4). Below ground biomass was recorded maximum in Sole Cowpea-Mustard which was 136.11, 133.73 and 136.36 percent higher than Gamhar + Arhar in 2016–17, 2017–18 and in pooled data, respectively.

**Table 4:** Above ground, below ground, total biomass and carbon stock of intercropsunder gamhar based agroforestry system.

Treatments	Above ground biomass (t ha^−1^)			Below ground biomass (t ha^−1^)			Total biomass (t ha^−1^)			Carbon stock (t ha^−1^)		
	2016–17	2017–18	Pooled	2016–17	2017–18	Pooled	2016–17	2017–18	Pooled	2016–17	2017–18	Pooled
Gamhar + Arhar	6.61 ± 0.20	7.20 ± 0.55	6.90 ± 0.110	0.72 ± 0.08	0.83 ± 0.12	0.77 ± 0.492	7.32 ± 0.28	8.03 ± 0.49	7.68 ± 0.526	3.29 ± 0.13	3.61 ± 0.22	3.45 ± 0.237
Gamhar + Cowpea-Mustard	9.31 ± 1.30	11.46 ± 1.67	10.39 ± 0.352	1.41 ± 0.28	1.69 ± 0.41	1.55 ± 1.786	10.72 ± 1.59	13.15 ± 2.02	11.93 ± 2.098	4.82 ± 0.71	5.92 ± 0.91	5.37 ± 0.944
Gamhar + Greengram-Mustard	9.53 ± 1.09	11.59 ± 0.39	10.56 ± 0.248	1.44 ± 0.25	1.51 ± 0.30	1.47 ± 1.349	10.96 ± 1.25	13.11 ± 0.09	12.04 ± 1.417	4.93 ± 0.56	5.89 ± 0.04	5.42 ± 0.638
*Sole Gamhar	–	–	–	–	–	–	–	–	–	–	–	–
Sole Arhar	7.33 ± 0.44	7.96 ± 0.55	7.65 ± 0.102	0.83 ± 0.05	0.94 ± 0.12	0.89 ± 0.567	8.16 ± 0.44	8.91 ± 0.57	8.53 ± 0.612	3.67 ± 0.20	4.00 ± 0.26	3.84 ± 0.275
Sole Cowpea-Mustard	10.71 ± 0.54	12.70 ± 0.18	11.70 ± 0.201	1.70 ± 0.21	1.94 ± 0.13	1.82 ± 1.150	12.41 ± 0.74	14.64 ± 0.29	13.53 ± 1.323	5.58 ± 0.33	6.59 ± 0.13	6.09 ± 0.595
Sole Greengram-Mustard	11.81 ± 1.07	14.04 ± 0.61	12.92 ± 0.425	1.18 ± 0.09	1.91 ± 0.20	1.54 ± 1.446	12.99 ± 1.10	15.95 ± 0.78	14.47 ± 1.830	5.85 ± 0.49	7.18 ± 0.35	6.51 ± 0.824
SEm ±	0.51	0.42	0.33	0.11	0.15	0.09	0.60	0.51	0.39	0.27	0.23	0.17
CD (*p* = 0.05)	1.62	1.34	0.98	0.36	0.47	0.27	1.91	1.60	1.16	0.86	0.72	0.52
CV (%)	9.66	6.80	8.15	16.44	17.61	17.22	10.07	7.18	8.53	10.07	7.18	8.53

*Sole Gamhar not included in statistical analysis.

Carbon sequestration, carbon credit and carbon price of crops were significantly affected by treatments (Table 5). Carbon sequestration, carbon credit as well as carbon price was maximum in Sole Greengram-Mustard which was 77.48, 77.48 and 77.47 percent higher in 2016–17, 98.64, 98.64 and 98.65 percent higher in 2017–18, and 88.62, 88.62 and 88.52 percent higher in pooled data, respectively as compared to the Gamhar + Arhar.

**Table 5:** Carbon sequestration, carbon credit and carbon price of intercrops under gamhar based agroforestry system.

Treatments	Carbon sequestration(t ha^−1^)			Carbon credit (No unit)			Carbon price($)		
	2016–17	2017–18	Pooled	2016–17	2017–18	Pooled	2016–17	2017–18	Pooled
Gamhar + Arhar	12.08 ± 0.47	13.25 ± 0.81	12.66 ± 0.869	12.08 ± 0.47	13.25 ± 0.81	12.66 ± 0.869	241.60 ± 0.47	265.00 ± 0.81	253.20 ± 0.869
Gamhar + Cowpea-Mustard	17.69 ± 2.62	21.70 ± 3.33	19.69 ± 3.462	17.69 ± 2.62	21.70 ± 3.33	19.69 ± 3.462	353.80 ± 2.62	434.00 ± 3.33	393.80 ± 3.462
Gamhar + Greengram-Mustard	18.09 ± 2.06	21.63 ± 0.15	19.86 ± 2.339	18.09 ± 2.06	21.63 ± 0.15	19.86 ± 2.339	361.80 ± 2.06	432.60 ± 0.15	397.20 ± 2.339
*Sole Gamhar	–	–	–	–	–	–	–	–	–
Sole Arhar	13.46 ± 0.72	14.70 ± 0.94	14.08 ± 1.010	13.46 ± 0.72	14.70 ± 0.94	14.08 ± 1.010	269.20 ± 0.72	294.00 ± 0.94	281.60 ± 1.010
Sole Cowpea-Mustard	20.48 ± 1.22	24.16 ± 0.48	22.32 ± 2.183	20.48 ± 1.22	24.16 ± 0.48	22.32 ± 2.183	409.60 ± 1.22	483.20 ± 0.48	446.40 ± 2.183
Sole Greengram-Mustard	21.44 ± 1.81	26.32 ± 1.29	23.88 ± 3.021	21.44 ± 1.81	26.32 ± 1.29	23.88 ± 3.021	428.80 ± 1.81	526.40 ± 1.29	477.60 ± 3.021
SEm ±	1.00	0.84	0.65	1.00	0.84	0.65	20.00	16.80	13.00
CD (*p* = 0.05)	3.15	2.65	1.92	3.15	2.65	1.92	63.00	53.00	38.40
CV (%)	10.07	7.18	8.53	10.07	7.18	8.53	10.07	7.18	8.53

*Sole Gamhar not included in statistical analysis.

### Estimation of soil carbon stock, carbon sequestration, carbon credit and carbon price

Carbon stock, carbon sequestration, carbon credit and carbon price in soil depth 0–30 cm were significantly affected by treatments (Table 6). Carbon stock, carbon sequestration, carbon credit as well as carbon price was maximum in Gamhar + Arhar which was 138.10, 136.64, 136.64 and 136.80 percent higher than Sole Gamhar in 2016–17, 537.50, 542.75, 542.75 and 541.59 percent higher in 2017–18, and 348.53, 352.23, 352.23 and 351.75 percent higher in pooled data, respectively.

**Table 6:** Carbon stock, carbon sequestration, carbon credit and carbon price of soil profile depths 0–30 cm under gamhar based agroforestry system.

Treatments	Carbon stock (t ha^−1^) 0–30 cm			Carbon sequestration (t ha^−1^) 0–30 cm			Carbon credit (No unit) 0–30 cm			Carbon price($) 0–30 cm		
	2016–17	2017–18	Pooled	2016–17	2017–18	Pooled	2016–17	2017–18	Pooled	2016–17	2017–18	Pooled
Gamhar + Arhar	1.50 ± 0.08	4.59 ± 0.34	3.05 ± 1.71	5.49 ± 0.31	16.84 ± 1.24	11.17 ± 6.27	5.49 ± 0.31	16.84 ± 1.24	11.17 ± 6.27	109.80 ± 0.31	336.80 ± 1.24	223.40 ± 6.27
Gamhar + Cowpea-Mustard	1.18 ± 0.06	1.26 ± 0.26	1.22 ± 0.18	4.34 ± 0.22	4.62 ± 0.97	4.48 ± 0.64	4.34 ± 0.22	4.62 ± 0.97	4.48 ± 0.64	86.80 ± 0.22	92.40 ± 0.97	89.60 ± 0.64
Gamhar + Greengram-Mustard	1.19 ± 0.01	1.33 ± 0.11	1.26 ± 0.10	4.37 ± 0.02	4.88 ± 0.42	4.63 ± 0.39	4.37 ± 0.02	4.88 ± 0.42	4.63 ± 0.39	87.40 ± 0.02	97.60 ± 0.42	92.60 ± 0.39
Sole Gamhar	0.63 ± 0.10	0.72 ± 0.06	0.68 ± 0.09	2.32 ± 0.37	2.62 ± 0.24	2.47 ± 0.32	2.32 ± 0.37	2.62 ± 0.24	2.47 ± 0.32	46.40 ± 0.37	52.40 ± 0.24	49.40 ± 0.32
Sole Arhar	0.79 ± 0.20	2.20 ± 0.34	1.50 ± 0.81	2.90 ± 0.75	8.06 ± 1.26	5.48 ± 2.97	2.90 ± 0.75	8.06 ± 1.26	5.48 ± 2.97	58.00 ± 0.75	161.20 ± 1.26	109.60 ± 2.97
Sole Cowpea-Mustard	0.68 ± 0.03	1.49 ± 0.34	1.09 ± 0.49	2.49 ± 0.11	5.47 ± 1.24	3.98 ± 1.81	2.49 ± 0.11	5.47 ± 1.24	3.98 ± 1.81	49.80 ± 0.11	109.40 ± 1.24	79.60 ± 1.81
Sole Greengram-Mustard	0.65 ± 0.04	1.10 ± 0.09	0.87 ± 0.31	2.38 ± 0.16	4.02 ± 0.35	3.20 ± 1.13	2.38 ± 0.16	4.02 ± 0.35	3.20 ± 1.13	47.60 ± 0.16	80.40 ± 0.35	64.00 ± 1.13
SEm ±	0.05	0.15	0.08	0.21	0.56	0.30	0.21	0.56	0.30	4.20	11.20	6.00
CD (*p* = 0.05)	0.17	0.47	0.24	0.65	1.75	0.88	0.65	1.75	0.88	13.00	35.00	17.60
CV (%)	10.61	14.80	14.73	10.70	14.82	14.77	10.70	14.82	14.77	10.68	14.81	14.75

### *Estimation of total (Tree* + *intercrops* + *Soil) carbon stock, carbon sequestration, carbon credit and carbon price*

Total (Tree + intercrops + Soil) carbon stock, carbon sequestration, carbon credit and carbon price of sole crops and gamhar based agroforestry system were significantly affected by treatments (Table 7). Carbon stock, carbon sequestration, carbon credit as well as carbon price was maximum in Sole Greengram-Mustard which was 812.86, 816.02, 816.02 and 817.00 percent higher than Sole Gamhar in 2016–17, 735.35, 733.52, 733.52 and 733.75 percent in 2017–18, and 772.62, 767.42, 767.42 and 768.11 percent in pooled data, respectively.

**Table 7:** Total carbon stock, carbon sequestration, carbon credit and carbon price of sole intercrops and gamhar based agroforestry system.

Treatments	Carbon stock (t ha^−1^) (Tree + intercrops + Soil)			Carbon sequestration (t ha^−1^) (Tree + intercrops + Soil)			Carbon credit (No unit) (Tree + intercrops + Soil)			Carbon price ($) (Tree + intercrops + Soil)		
	2016–17	2017–18	Pooled	2016–17	2017–18	Pooled	2016–17	2017–18	Pooled	2016–17	2017–18	Pooled
Gamhar + Arhar	4.86 ± 0.051	8.50 ± 0.411	6.68 ± 2.01	17.83 ± 0.185	31.16 ± 1.508	24.50 ± 7.367	17.83 ± 0.185	31.16 ± 1.508	24.50 ± 7.367	356.60 ± 0.185	623.20 ± 1.508	490.00 ± 7.367
Gamhar + Cowpea-Mustard	6.08 ± 0.667	7.50 ± 0.847	6.79 ± 1.03	22.29 ± 2.444	27.49 ± 3.104	24.89 ± 3.790	22.29 ± 2.444	27.49 ± 3.104	24.89 ± 3.790	445.80 ± 2.444	549.80 ± 3.104	497.80 ± 3.790
Gamhar + Greengram-Mustard	6.20 ± 0.557	7.55 ± 0.139	6.87 ± 0.82	22.73 ± 2.042	27.68 ± 0.508	25.21 ± 3.023	22.73 ± 2.042	27.68 ± 0.508	25.21 ± 3.023	454.60 ± 2.042	553.60 ± 0.508	504.20 ± 3.023
Sole Gamhar	0.70 ± 0.107	0.99 ± 0.063	0.84 ± 0.18	2.56 ± 0.393	3.64 ± 0.229	3.10 ± 0.659	2.56 ± 0.393	3.64 ± 0.229	3.10 ± 0.659	51.20 ± 0.393	72.80 ± 0.229	62.00 ± 0.659
Sole Arhar	4.46 ± 0.327	6.20 ± 0.109	5.33 ± 0.98	16.36 ± 1.199	22.75 ± 0.399	19.56 ± 3.591	16.36 ± 1.199	22.75 ± 0.399	19.56 ± 3.591	327.20 ± 1.199	455.00 ± 0.399	391.20 ± 3.591
Sole Cowpea-Mustard	6.26 ± 0.312	8.08 ± 0.457	7.17 ± 1.05	22.97 ± 1.143	29.63 ± 1.674	26.30 ± 3.868	22.97 ± 1.143	29.63 ± 1.674	26.30 ± 3.868	459.40 ± 1.143	592.60 ± 1.674	526.00 ± 3.868
Sole Greengram-Mustard	6.39 ± 0.536	8.27 ± 0.304	7.33 ± 1.10	23.45 ± 1.966	30.34 ± 1.113	26.89 ± 4.034	23.45 ± 1.966	30.34 ± 1.113	26.89 ± 4.034	469.00 ± 1.966	606.80 ± 1.113	537.80 ± 4.034
SEm ±	0.25	0.24	0.17	0.92	0.87	0.63	0.92	0.87	0.63	18.40	17.40	12.60
CD (*p* = 0.05)	0.77	0.73	0.50	2.84	2.70	1.86	2.84	2.70	1.86	56.80	54.00	37.20
CV (%)	8.74	6.17	7.26	8.74	6.17	7.26	8.74	6.17	7.26	8.74	6.17	7.26

### Estimation of total carbon emission, carbon sequestration and net emission

**Figure 1:**
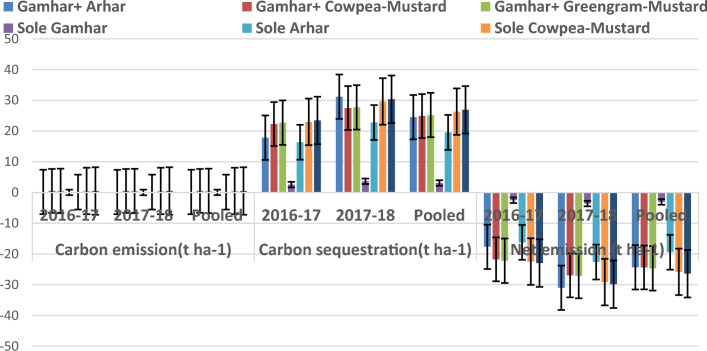
Carbon emission, carbon sequestration and net emission of sole intercrops and gamhar based agroforestry system.

The annual total (Tree + interrops + Soil) carbon emission, carbon sequestration and net emission in tonnes per hectare (t ha^-1^) has been presented in the Fig. 1. Total (Tree + interrops + Soil) carbon sequestration and net emission of sole intercrops and gamhar based agroforesrty system were significantly affected by treatments. Carbon emission was maximum in Gamhar + Cowpea-Mustard and Gamhar + Greengram-Mustard, (both being same because of same inputs used), which was 1284.21, 2218.18 and 1626.67 percent higher than Sole Gamhar in 2016–17, 2017–18 and in pooled data, respectively. In case of carbon sequestration, maximum was observed in Sole Green gram-Mustard which was 817.09, 733.69 and 768.11 percent higher than in Sole Gamhar 2016–17, 2017–18 and in pooled data, respectively. Net emission was recorded 811.55, 725.24 and 760.69 percent lower than Sole Gamhar 2016–17, 2017–18 and in pooled data, respectively. The negative value of the data indicates that carbon sequestration was more than carbon emission.

## Discussion

Total (tree + interrops + soil) carbon stock, carbon sequestration, carbon credit and carbon price of sole intercrops and gamhar based agroforestry system were significantly affected by the treatments. Carbon stock, carbon sequestration, carbon credit as well as carbon price were maximum in Sole Greengram-Mustard. All parameters were higher in sole intercrops than gamhar based agroforestry system because gamhar plants were very small (five months old) at the time of planting and the total dry biomass production was lower in trees than in intercrops. The height, diameter, basal area and volume of Gamhar after completing 12 years was lower under sole plantation compared to agri-silvicultural system^16^. Similar results have also been confirmed by^17^ observed that the cash flow analysis of the carbon trading neutral products reported substantial initial investments during the first 3 years of the project, while benefits are obtained after completing 4 years. Kumar et al.^18^ reported that the total tree biomass (3.707 t ha^−1^), carbon stock (1.597 t ha^−1^), carbon sequestration (5.862 t ha^−1^), carbon credit (5.86) and carbon price ($ 103.76) respectively were estimated at the age of two years of poplar tree-based agroforestry system. Tamang et al.^19^ studied the carbon sequestration potential of gamhar (*Gmelina arborea*) being higher compared to other tree species making it very suitable for reduction of atmospheric carbon (CO_2_e) under higher temperatures by implementing a planned for conservation of plant diversity. Tamang et al.^19^ reported that the total C stock of the ecosystem’s vegetation + soil C (0–30 cm) in the forested area was 275 t ha^−1^, equating to 37 t ha^−1^in the agricultural system alone, and results highlighted that agroforestry systems have the highest potential for C sequestration. Among the studied tree species, the soil carbon density and carbon sequestration potential (CSP) were found to be maximum (13.56 t ha^−1^ and 1.28 t ha^−1^ year^−1^) in *Gmelina arborea* followed by *Eucalyptus tereticornis*, *Cassia siamea*, and *Leucaena leucocephala*, respectively, depicting that these tree species have a stronger capacity to sequester and store carbon, making them suitable as atmospheric carbon reducers^20^. The above ground estimated carbon stock of *Gmelina arborea* approximately 13 Mg ha^−1^ or 47 Mg CO_2_e ha^−1^ in 9 months, making it a valuable and promising species for CO_2_ sequestration under the context of climate change^21^. Similar results and reasons have been also confirmed by^22,23^.

But several researchers have found that in long term basis agroforestry systems contains very high potential for enhancing total biomass, carbon sequestration, carbon credit, carbon trading, support local peoples, and strengthen the climate resilience in agriculture compared to sole cropping system. Because agroforestry have more carbon sequestration potential and lower carbon emission through trees, intercrops and soil; similar results have also been confirmed by Orwa et al.^24^ reported significantly lower net carbon emission ha^-1^ in agroforestry system (− 40.998 t ha^−1^) as compared to open farming (− 37.263 t ha^−1^) despite higher emission (1.052 t ha^−1^ as compared to 0.998 t ha^−1^ in open farming) due to more carbon sequestered by trees in the agroforestry system (42.049 t ha^−1^ as compared to 38.261 t ha^−1^ in open farming). Azeez et al.^25^ reported that in mustard field, the CO_2_ emission values ranging from 1.083 to 1.683 t C ha^−1^ were not significantly affected by the crop cultivation treatment. According to ^26^the average yearly GHG emissions ranged from 0.93 to 1.60 t CO_2_e ha^−1^ yr^−1^, which may be considered low when compared to other systems, probably due to adoption of agroforestry systems with reduced fuel inputs, land practices, machinery use and CO_2_ emissions. The common management practices in agroforestry systems, such as zero-tillage farming and optimal fertilizer/manure regimes can increase carbon sequestration while reducing carbon and other GHG emissions^27^. Hung et al.^28^ also reported that on different types of agroforestry systems, the total greenhouse gas emissions were 7.98, 4.25, 4.04 and 2.80 t CO_2_e ha^−1^. Similar results and reasons have been also confirmed by^29–32^.

## Conclusion

This study concludes that the total (tree + intercrops + soil) above ground, below ground, total biomass production, carbon stock, carbon sequestration, carbon credit and carbon trading were found higher in the treatment Sole Greengram-Mustard as compared to all treatments. Whereas carbon emission was lower in tree components of all the system and kept on declining in the successive years. Gmahar is a fast-growing tree species, therefore agroforestry based on this tree have high potential of carbon sequestration and lower carbon emission in long term basis. It can be a tool to increase the tree coverage, reducing 1 billion tonnes of carbon emissions by 2030, enhance carbon sequestration, carbon credit, carbon trading, support local peoples, and strengthen the climate resilient agriculture thereby supporting global SDGs and climate change mitigation and adaptation. It will also support India’s national goals, international commitments related to Climate initiatives regarding ‘Panchamrita’, ‘Mission LiFE’, TOFI programme and net zero carbon emissions goal by 2070.

## Materials and methods

### Site description and experimental setup

**Figure 2:**
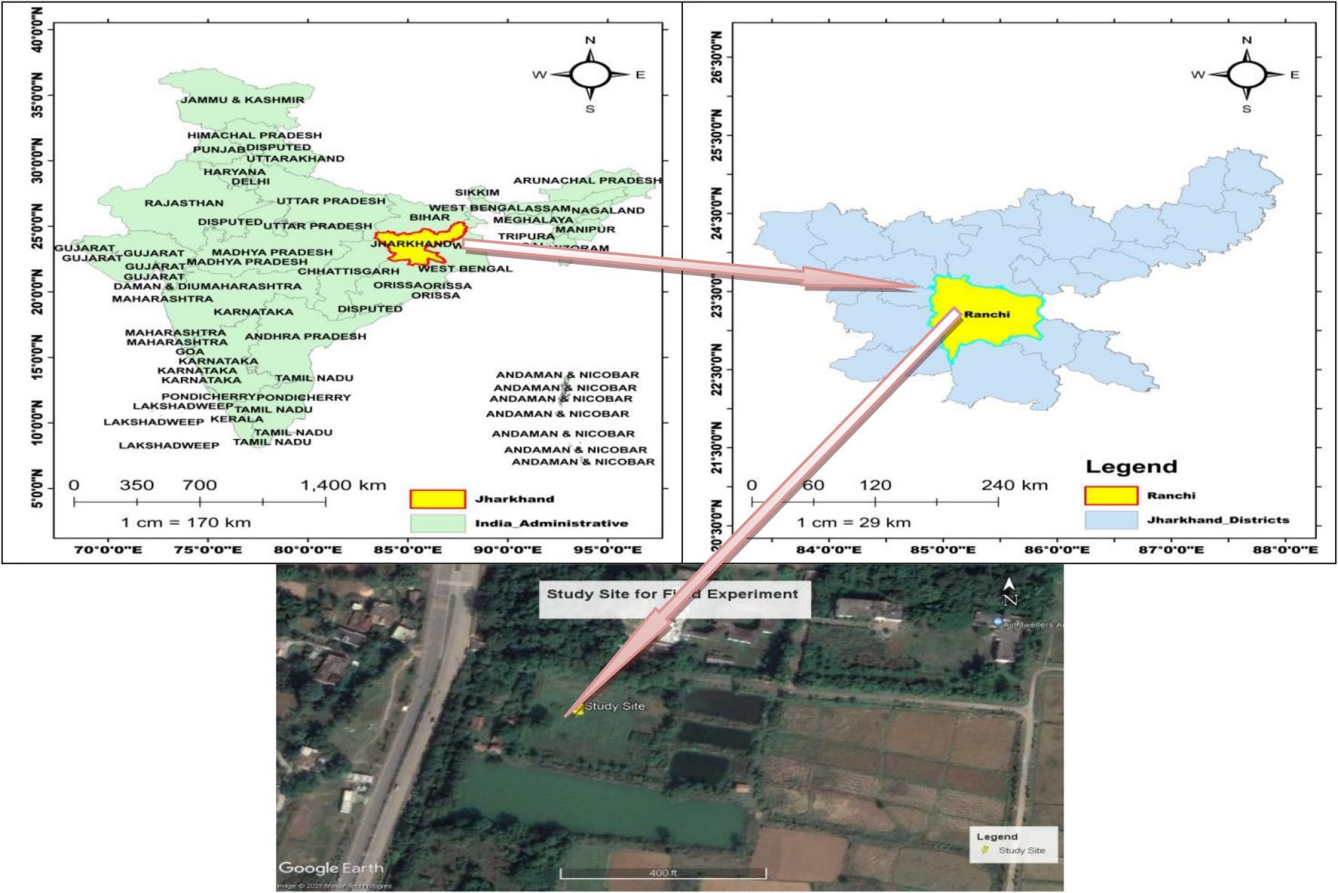
Location of the experimental site.

The research experiment was conducted during monsoon and winter season of 2016–17 and 2017–18 at the field experimental site close to Faculty of Forestry, Birsa Agricultural University, Kanke, Ranchi, Jharkhand, India. It is a national government institution. The research experimental site is located between 23°26′54.6′′ N to 23°26′55.0′′ N Latitude and 85°18′53.0′′ E to 85°18′53.7′′ E longitudes and at an altitude of 625 m above the mean sea level (MSL). It is the eastern section part of the Deccan plateau region and comes under the agro-climatic zone (Zone VII) of the India known as Eastern Plateau and Hill Region. The experimental site is shown in Fig. 2.

The entire experiment field site was laid out as per plan of All India coordinate research project on agroforestry under ICAR, govt. of India. The planted gamhar tree (*Gmelina arborea* Roxb.) and four different intercrops are Arhar (*Cajanus cajan*), Cowpea (*Vigna unguiculata*), Greengram (*Vigna radiata*), Mustard (*Brassica juncea*) under gamhar based agroforestry system and in sole (open) conditions. The field experiment design adopted was randomized block design (RBD) with seven treatments and five replications. They were: T_1_: Gamhar + Arhar, T_2_: Gamhar + Cowpea-Mustard, T_3_: Gamhar + Greengram-Mustard, T_4_: Sole Gamhar, T_5_: Sole Arhar, T_6_: Sole Cowpea-Mustard, T_7_: Sole Greengram-Mustard. The experimental field plot size was 24 × 7.5 m^2^ and nursery raised five months old quality seedlings of uniform size gamhar tree were transplanted in the field and collection of plant material, complies with relevant institutional, national, and international guidelines and legislation on June, 2016; in pits of 45 cm × 45 cm × 45 cm size at spacing of 8 m × 2.5 m (500 plants ha^-1^) and intercrops being cultivated during monsoon and winter season of 2016–2017 and 2017–2018.

### Estimation of standing tree biomass

#### Above ground biomass of tree

Gamhar (*Gmelina arborea*) trees were measured for their height from ground level to top of the trees and girth at collar diameter. The volume of stem was calculated with the help of girth and height. Standing tree volume of stem was calculated by the quarter girth formula:

1 $${\text{Total stem volume }}\left( {{\text{m}}^{{3}} } \right) \, \left( {{\text{V}}_{{{\text{ob}}}} } \right) \, = \, \left( {{\text{G}}/{4}} \right)^{{2}} \times {\text{ H}}$$

where, V_ob_, G and H represent volume of tree over bark, girth of tree and height of the tree, respectively.

The above ground stem biomass was calculated by the formula:

2 $${\text{Biomass }} = {\text{ volume }} \times {\text{ specific gravity of wood }}\left( {{53}0{\text{ kg m}}^{{ - {3}}} {\text{for gamhar tree given by}}^{{{33},{34}}} } \right).$$

Gamhar trees were then divided into individual components such as stem, branches and leaves. The branches were counted in the standing tree and detached ten different sizes of reference branches from the standing tree by random selection with the help of cutting scissors. These branches contained different sizes of leaves. The leaves were removed from branch. The fresh weights were determined for branches and their leaves by using a balance. The entire samples (branch and leaf) were packed in the bags and brought into laboratory for drying in oven at 72 °C for 48 h. The oven dry weight of each sample was estimated. The dried weights of collected branches and leaves were used for estimation of standing biomass of tree. Total above ground biomass was computed by summing the biomass of stem, branch and leaves components.

#### Below ground biomass of tree

Below ground biomass contains the root of the gamhar tree. The below ground biomass was estimated by using a simple default value of 25 percent (for hardwood species) of the total above ground biomass as suggested by the^35^.

3 $${\text{Below ground biomass }} = {\text{ Above ground biomass }} \times \, 0.{25}$$

#### Total biomass of tree

The total biomass was estimated by adding biomass of all the components (above ground and below ground).

4 $${\text{Total biomass of tree}} = {\text{Above ground biomass }}({\text{stem}},{\text{ branch and leaves}}) \, + {\text{Below ground biomass }}\left( {{\text{root}}} \right)$$

### Estimation of intercrops biomass

#### Above ground biomass of intercrops

Above ground biomass of intercrops (arhar, cowpea, greengram and mustard) were recorded for per meter square. All plants were uprooted from the ground level and divided into two components viz., above ground (stem, branches, leaves and pod or siliqua) and below ground (root). The separated components were oven dried at 70 ± 2 °C in an electric oven till constant weight. The oven dry weight of intercrop samples measured on digital pan balance. Dry intercrop yields the same as above ground biomass.

#### Below ground biomass of intercrops

Below ground biomass of intercrops (arhar, cowpea, greengram and mustard) were recorded per meter square from net plot at harvesting stages. Below ground biomass contains roots of the intercrops. Similarly, dry intercrop root yields the same as below ground biomass (root).

#### Total biomass of intercrops

5 $${\text{Total biomass of intercrops}} = {\text{Above ground biomass }}\left( {{\text{stem}},{\text{ branches}},{\text{ leaves and pod or siliqua}}} \right) \, + {\text{Below ground biomass }}\left( {{\text{root}}} \right)$$

#### *Total biomass (tree* + *intercrops)*

The total biomass was recorded on above ground biomass (tree + intercrops) and below ground (tree + intercrops) of gamhar and intercrops (arhar, cowpea, green gram and mustard) under sole cropping system and gamhar based agroforestry system.

6 $${\text{Total biomass }}\left( {{\text{tree}} + {\text{intercrops}}} \right) = {\text{Above ground biomass }}\left( {{\text{tree}} + {\text{intercrops}}} \right) + {\text{Below ground biomass }}\left( {{\text{tree}} + {\text{intercrops}}} \right)$$

### Estimation of total carbon stock

#### Carbon stock in tree

Carbon stock was derived from above ground and below ground biomass by assuming that nearly 50% of the biomass is made up by carbon^35–37^. So, the carbon stock for tree was determined by multiplying total biomass (above ground + below ground) with carbon conversion factor of 0.50

7 $${\text{Carbon stock of tree}} = {\text{ Total dry biomass }} \times \, 0.{5}0$$

#### Carbon stock in intercrops

The carbon stock in herbs and shrub species was determined by multiplying total biomass (above ground + below ground biomass) with carbon conversion factor of 0.45^38,39^.

8 $${\text{Carbon stockof intercrops }} = {\text{ Total dry biomass }} \times \, 0.{45}$$

#### Carbon stock in soil

According to^40^ the capacity of carbon storage in soil is higher than vegetation and atmosphere; and giving it a play major role in global carbon sequestration^41^. The carbon stock in soil was calculated by the formula as follows^42,43^.

9 $${\text{Soil carbon stock }}\left( {{\text{t ha}}^{{ - {1}}} } \right) = {\text{Soil organic carbon }}\% \, \times {\text{ Soil sampling depth }}\left( {{\text{cm}}} \right) \, \times {\text{ Bulk density }}\left( {{\text{g cm}}^{{{-}{3}}} } \right)$$

#### Total carbon stock

The total carbon stock was estimated by adding of all the components (tree + intercrops + soil).

10 $${\text{Total carbon stock}} = {\text{Total tree carbon stock}} + {\text{Total intercrops carbon stock}} + {\text{Total soil carbon stock}}$$

### Estimation of total carbon sequestration

Carbon sequestration is the procedure of capturing and storing of atmospheric carbon dioxide by the plant. Total carbon sequestration was obtained by addition of carbon sequestrated by total carbon stock (total tree carbon stock + total intercrops carbon stock + total soil carbon stock). The estimated total carbon stocks were converted into carbon sequestration, multiplied by 44/12 or 3.666^44^.

11 $${\text{Carbon sequestration }} = {\text{ Total carbon stock }} \times { 3}.{666}$$

### Estimation of total carbon credit

The total carbon credit or certified emission reduction (CER) is the reduction/sequestration of one tonnes of atmospheric carbon emission. The one tonnes of sequestered carbon dioxide in the form of plant biomass is equal to one carbon credit or CER^18^. So, total carbon credits of gamhar based agroforestry system were estimated from the carbon equivalent values of retained total tree and intercrops biomass (Supplementary Material).

### Carbon trading

The price of carbon credit was found very variable among different countries, so price taken from the international market. The price of one carbon credit or CER in Indian Rupees is about ₹ 1500 or $ 20^45,46^.

### Carbon emission

The emission of carbon (CO_2_-e) was calculated by the software “Green House Gases Estimation Tool for Integrated Farming System Models” developed by the ICAR-Indian Institute for Farming System Research, Modipuram, Meerut, Uttar Pradesh, India All the inputs used during research period, viz. energy/fuel used for different practices of field preparation, application of water for irrigation, fertilizers, organic manures, herbicide, pesticide and any other farm machinery used for harvest of intercrops, etc. were in use into account as for the estimation of carbon emission. The net carbon emission was then estimated by subtracting the total carbon sequestered in the research field from total carbon emission from the experimental field.

### Statistical analysis

The obtained data was analyzed using standard statistical procedure for Randomized Block Design (RBD) with the help of computer applying IBM-SPSS statistical analysis of variance (ANOVA) technique. Standard error of mean (SEm ±) and C.V. was computed in each case by using the critical difference (C.D.) at 5 percent probability level to test the effects of treatment.

### Supplementary Information

Supplementary Information.

## Data Availability

The original contributions presented in the study are included in the article/supplementary material, further inquiries can be directed to the corresponding author.
